# Screening and evaluating of long noncoding RNAs in the puberty of goats

**DOI:** 10.1186/s12864-017-3578-9

**Published:** 2017-02-14

**Authors:** Xiaoxiao Gao, Jing Ye, Chen Yang, Kaifa Zhang, Xiumei Li, Lei Luo, Jianping Ding, Yunsheng Li, Hongguo Cao, Yinghui Ling, Xiaorong Zhang, Ya Liu, Fugui Fang, Yunhai Zhang

**Affiliations:** 1 Anhui Provincial Laboratory of Animal Genetic Resources Protection and Breeding, College of Animal Science and Technology, Anhui Agricultural University, 130 Changjiang West Road, Hefei, Anhui 230036 China; 2 Anhui Provincial Laboratory for Local Livestock and Poultry Genetic Resource Conservation and Bio-Breeding, 130 Changjiang West Road, Hefei, Anhui 230036 China; 3 Department of Animal Veterinary Science, College of Animal Science and Technology, Anhui Agricultural University, 130 Changjiang West Road, Hefei, Anhui 230036 China

**Keywords:** LncRNA, Puberty, Goat, Hypothalamus, Transcriptome

## Abstract

**Background:**

Long noncoding RNAs (lncRNAs) are involved in regulating animal development, however, their function in the onset of puberty in goats remain largely unexplored. To identify the genes controlling the regulation of puberty in goats, we measured lncRNA and mRNA expression levels from the hypothalamus.

**Results:**

We applied RNA sequencing analysis to examine the hypothalamus of pubertal (case; *n* = 3) and prepubertal (control; *n* = 3) goats. Our results showed 2943 predicted lncRNAs, including 2012 differentially expressed lncRNAs, which corresponded to 5412 target genes. We also investigated the role of lncRNAs that act *cis* and *trans* to the target genes and found a number of lncRNAs involved in the regulation of puberty and reproduction, as well as several pathways related to these processes. For example, oxytocin signaling pathway, sterol biosynthetic process, and pheromone receptor activity signaling pathway were enriched as Kyoto Encyclopedia of Genes and Genomes (KEGG) or gene ontology (GO) analyses showed.

**Conclusion:**

Our results clearly demonstrate that lncRNAs play an important role in regulating puberty in goats. However, further research is needed to explore the functions of lncRNAs and their predicted targets to provide a detailed expression profile of lncRNAs on goat puberty.

**Electronic supplementary material:**

The online version of this article (doi:10.1186/s12864-017-3578-9) contains supplementary material, which is available to authorized users.

## Background

Puberty is a pivotal stage in female goat development. It marks the first occurrence of ovulation and the onset of reproductive capability [1]. The mechanism of puberty onset is complex and thought to be associated with environmental factors, neuroendocrine factors, genetic factors and their interactions. In general, the secretion of gonadotropin-releasing hormone (GnRH) is considered a crucial factor in puberty onset for goats [2]. A popular view is that during the prepubertal period, secreting neurons suffer persistent trans-synaptic inhibition. This means that GnRH secretions increase as long as this inhibition is eliminated, which leads to puberty [2]. However, these influences are based on substantial genetic control [3].

It was previously reported that the initiation of puberty in female rats is regulated by epigenetic mechanism of transcriptional repression [4], whereby epigenetic control was composed of several mechanisms. Two well established mechanisms include: modification of chromatin and chemical modification of the DNA (including DNA methylation and hydroxymethylation). Non-coding RNA is the most recently unveiled mechanism of epigenetic control, which affords epigenetic information by lncRNAs or microRNAs [5]. Broadly, lncRNAs are known as transcripts greater than 200 nt in length that do not appear to code proteins [6].

During the past decades of transcriptome studies, multiple lncRNAs have been discovered, such as *Xist* and *H19*. The advent of RNA-seq has been a powerful tool in exploring and quantifying lncRNAs [7], which has led to the identification of many more lncRNAs that await functional validation. Most identified lncRNAs have primarily originated from human and mouse studies [8, 9]. Recent studies in bovine [10–12] and porcine species [13, 14] have enriched the mammalian lncRNAs databases, providing a promising future for further lncRNAs studies.

LncRNAs have been shown to participate in the regulation of transcriptional and post-transcriptional control [15]. In recent years, lncRNAs have proven to play roles in lactation, ovary development, and embryo and sperm maturation. Therefore, we inferred that the onset of goat puberty is also regulated by lncRNAs. In this study, we applied RNA-seq and investigated the expression profiles of mRNA and lncRNAs in pubertal and prepubertal goats to explore the association of lncRNAs with the onset of puberty.

## Methods

### Preparation of animals and tissues

This study was authorized and endorsed by the Animal Care and Use Committee of Anhui Agricultural University. We housed three, prepubertal (aged 2.5-3.0 months) and three, pubertal (aged 4.5-5.0 months) female Anhuai goats under the same conditions on a farm in Anhui Province, China. We determined puberty goats in studied femal by male goats detecting estrus and the appearance changes of vulva [16]. The average weight of pubertal goats was 16.17 kg compared with the prepubertal goats 8.30 kg, and the average weight of the pubertal goats’ ovary was 0.76 g compared with the prepubertal goats 0.30 g. The animals were deeply anesthetized by intravenous administration of 3% pentobarbital sodium (30 mg/kg; Solarbio, P8410, China) and sacrificed by exsanguination in a healthy physiological stage when pubertal goats were in the late follicular phase. Hypothalamic tissues were surgically removed, and frozen in liquid nitrogen immediately. These tissues were stored at −80 °C until the RNA extraction [17].

### RNA sequencing and quality control

We isolated total RNA from goat hypothalamus using TRIzol Reagent (Invitrogen, Carlsbad, CA, USA), according to the standard extraction protocol. The contamination and degradation of RNA was detected by 1% agarose gels. The purity of RNA was monitored using the NanoPhotometer® spectrophotometer (Implen, Los Angeles, CA, USA). We measured the concentration of RNA using Qubit® RNA Assay Kit in Qubit® 2.0 Flurometer (Life Technologies, Carlsbad, CA, USA). The integrity of RNA was monitored as previous reported [18]. We used 3 μg RNA per sample for the RNA sample preparations. Firstly, ribosomal RNA in total RNA was removed [19], and then the residue was cleaned up by using ethanol precipitation. Then the libraries whith high strand-specificity for sequencing was generated [19], following manufacturer’s recommendations. Then the process was followed as previously described [20]. Illumina Hiseq 4000 platform was adopted on sequencing and 150 bp paired-end reads were generated. Raw reads were dealt with in-house perl scripts. The reads with more than 10% unknown bases, reads containing adapter and reads with more than 50% of low-quality bases (whose Phred scores were < 5%) were removed, yielding only the clean reads. Meanwhile, the quality of clean reads (Q20, Q30, and GC content) were detected. All the following analyses were based on high quality clean reads.

### Transcriptome assembly

We used a GTF file (ftp://ftp.ncbi.nlm.nih.gov/genomes/Capra_hircus/GFF/) with the annotation of the goat genome. Index of the reference genome was created by Bowtie v2.0.6 [21, 22] and then we aligned paired-end clean reads to the reference genome using TopHat v2.0.9 [23]. The mapped reads of each sample were assembled by both Scripture (beta2) [24] and Cufflinks (v2.1.1) [25, 26] in a reference-based approach. Both methods determined exons connectivity by spliced reads. Scripture ran using default parameters, while Cufflinks ran with min-frags-per-transfrag = 0’ and–library-type fr-firststrand’. Other parameters were set as default.

### Expression and coding potential analysis of transcripts

Gene expression was calculated using FPKMs of transcripts in each sample [27]. We confirmed differential expression in gene expression data using Cuffdiff as it based on the negative binomial distribution provides statistical routines [25]. Transcripts with a *P* < 0.05 were assigned as significantly differentially expressed between two groups. We used three analytic tools, including Coding-Non-Coding-Index (CNCI; v2) [28], Coding Potential Calculator (CPC; 0.9-r2) [29], Pfam Scan (v1.3) [30] to screen out candidate lncRNAs. CNCI (v2) profiles distinguished protein-coding and non-coding sequences effectively by adjoining nucleotide triplets, which was independent of known annotations. CPC (0.9-r2) was mainly used to detect the extent and quality of the Open Reading Frames (ORF) in a transcript and discover the sequences in known protein database, clarifying the coding and non-coding transcripts. Each transcript was translated in all three possible frames, then any of the known protein family was identified by Pfam Scan (v1.3) in the Pfam database (release 27; adopted both Pfam A and Pfam B). The coding potential of transcripts predicted by any of the three tools above were filtered out (non-annotated transcriptional activity by identifying novel transcripts), and those without coding potential were our candidate lncRNAs for further analysis.

### Target gene prediction and functional enrichment analysis

The *cis* role refers to the lncRNA acting on neighboring target genes [31, 32]. To predict the *cis*-regulated target genes of lncRNAs, we screened protein-coding genes as potential targets 10 K/100 K upstream and downstream of lncRNAs andanalyzed their function. The *trans* role refers to the coexpression relationship between lncRNAs and mRNA. Expression levels of lncRNAs and mRNAs were calculated for Pearson’s correlation coefficients by custom scripts (r > 0.95 or r < −0.95). The target genes of lncRNAs were performed functional enrichment analysis by clustering the genes from various samples using the DAVID platform [33]. The significance was described as a *P*-value, measured by the EASE score (*P* < 0.05 was considered significant).

### Quantitative real-time PCR

We have validated the RNA-seq data by selected eight lncRNAs, two novel transcripts, and two target genes to investigate the expression patterns in the samples using qRT-PCR. Werepeated the qRT-PCR experiments three times per sample on expression from the same hypothalamic tissues of three pre vs. three pubertal goats. We designed primers online using Primer5 software and evaluated using BLAST at NCBI. A list of the primer sequences is shown in Table 1. We performed qRT-PCR using SYBR green (Vazyme, China) method. Expression levels of genes were quantified through the cycle threshold (Ct) values and evaluated as 2^-ΔΔCT^. The data of expression was normalized to *β-action.*

**Table 1:** qRT-PCR primer and size of the amplification products of the target and housekeeping genes

Gene	Forward primer, 5’-3’	Reverse primer, 5’-3’	Product size, (bp)
XLOC_957527	ACACGACCAGAACATCAG	AATCACAGGAGAAGAGTAGG	162
XLOC_910648	TGCCATCCAGCCATCTCATC	CACACACCACAGTTCCTTTACC	175
XLOC_1101518	CTCCTGGGCTACCGAATGT	GCGGCTGTGAACTAAATGG	172
XLOC_1276445	TCGCTCCGTCTTCACCTAC	CGTTGCTCCATCACCCTTG	100
XLOC_2056339	CCTGTTGTTGGAATCACTC	CTCTTATGCCTCGGATGG	184
XLOC_960044	CAAGGAGTCGCACGCTAC	CTCTTACGCCTCTGAATCGG	128
XLOC_032671	TGATGCCAAGAGGTAGCC	TTATACAGACAGTGAAAGAGAGG	180
XLOC_688924	ATCTCCACTCTACAAACCTATACC	ACTCTCAAAGGGAAGCCAATG	142
Novel_000476	TTACCTACTACCATCCTTCAC	ATCAGCAGAACCAGAACC	178
Novel_000453	GAAGTGTCGTCTGGAGATTACC	TGTTGAGTGAGTCCTGTATTACC	181
CD38	CTACTGCCTTCTTCTGTG	TTCTGCTTCTGGAATACG	185
GnRH1	CTAATCCTGCTGACTTTCTGTG	ACCTCTTTGGCTATCTCTTGG	127
β-actin	CGTGACATCAAGGAGAAG	GAAGGAAGGCTGGAAGAG	171

### GO and pathway analysis

In this study, Gene Ontology (GO) enrichment [34] analysis of targets was performed by the GOseq R package and corrected by *P* (*P* < 0.05 were considered significantly enriched). Pathway analysis is a functional analysis in KEGG (http://www.genome.jp/kegg) pathways [35]. We evaluated the statistical enrichment of lncRNAs target genes or differential expression genes in KEGG pathways using KOBAS [36] software.

### Statistical analysis

We performed further analysis of RNA-seq data and graphical representations using the statistical R package (R, Auckland, NZL), adopting multiple testing and *P* corrections. We applied SPSS 17.0 software package (SPSS, Chicago, IL, USA) to analyze the qRT-PCR data. Differential expression levels of genes were calculated by independent-samples *t*-test between prepubertal and pubertal goats. Significance of data was defined as *P* < 0.05.

## Results

### Identification of lncRNAs

**Fig. 1:**
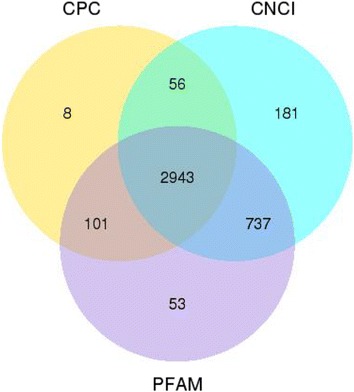
Screening of candidate lncRNAs in hypothalamus transcriptome. The coding potential of lncRNAs were analyzed by three tools (CPC, CNCI and PFAM)

We used pubertal and prepubertal goats to perform RNA-seq analysis from the hypothalamus of six female Anhuai goats. In total, 774,998,560 raw reads were produced under the Illumina HiSeq 4000 platform. We obtained 636,544,196 reads maped to goat reference genome after discarding low-quality sequences and adaptor sequences. The percentage of mapped reads among clean reads in each library ranged from 80.45% - 84.32% (Additional file 1). After the analysis of coding potential using the CNCI, CPC and Pfam-scan software, we identified 2943 lncRNAs (Fig. 1), including 2426 large intergenic noncoding RNAs, 217 anti-sense_lncRNAs, and 300 intronic_lncRNAs.

### Genomic features of lncRNAs

**Fig. 2:**
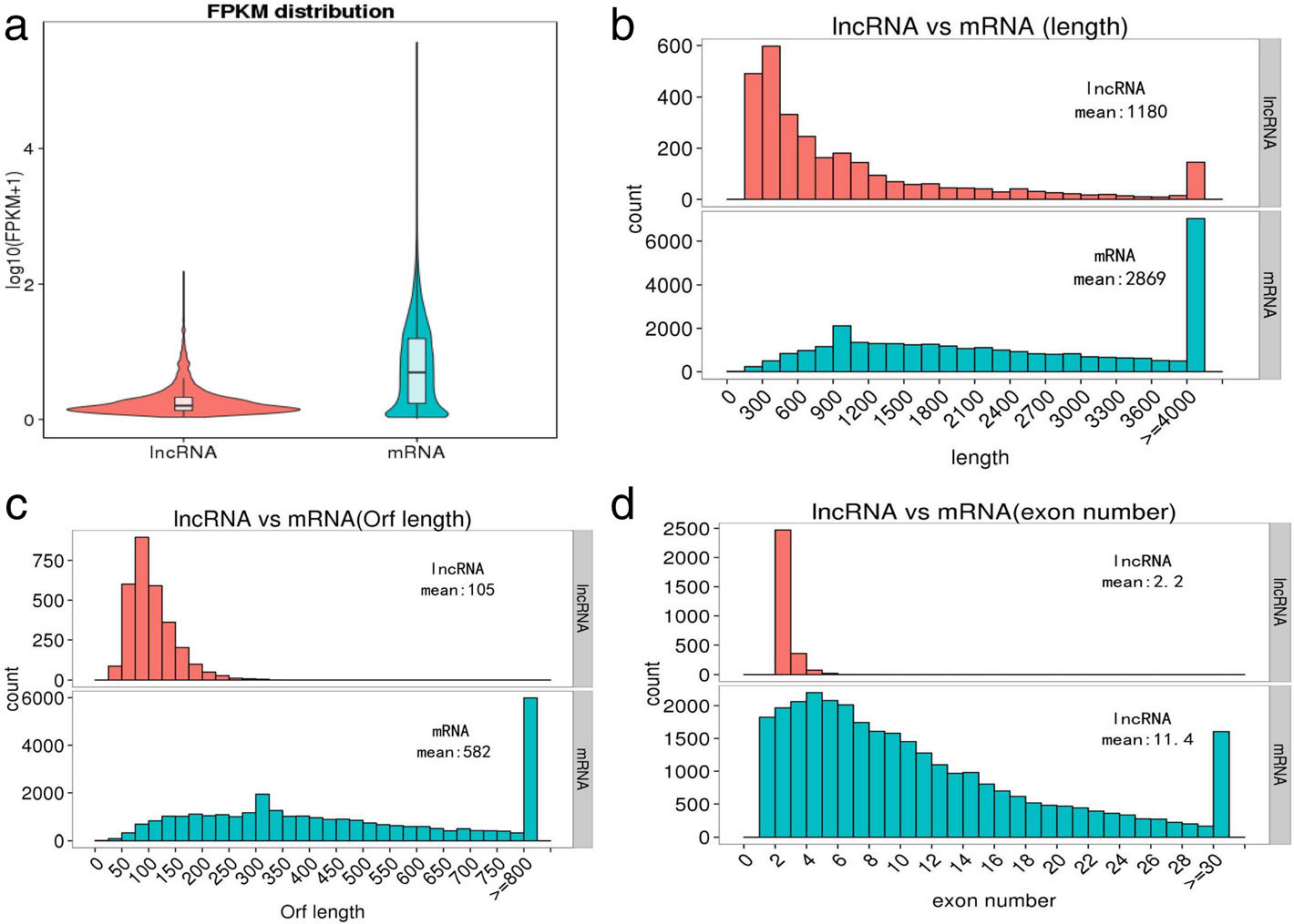
The comparison of features between predicted lncRNAs and mRNA. **a** Expression of lncRNAs and mRNA. **b** Length distribution of 2943 predicted lncRNAs and 30162 coding transcripts. **c** ORF length distribution of lncRNAs and coding transcripts. **d** Exon number distribution of lncRNAs and coding transcripts

Overall, we observed a lower expression of lncRNAs compared with mRNA (Fig. 2a). The mean length of lncRNAs in our dataset was 1180 nt, and the mean mRNA length was 2869 nt (Fig. 2b). Furthermore, we detected an ORF mean length of 105 nt for our lncRNAs, which tended to be shorter than protein coding genes (Fig. 2c). We also found lncRNAs contained fewer exons than mRNA (Fig. 2d).

### Differential expression cluster analysis

**Fig. 3:**
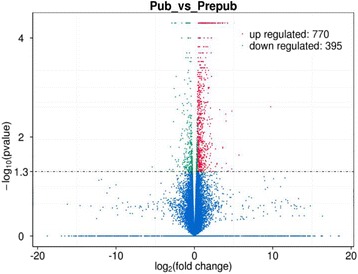
Volcano plots of differential expression transcripts. X-axis is fold change (log 2) and Y-axis is *P* (−log 10). Red points indicate up-regulated (X axis > 0) transcripts; green points indicate down-regulated (X axis < 0) transcripts

**Fig. 4:**
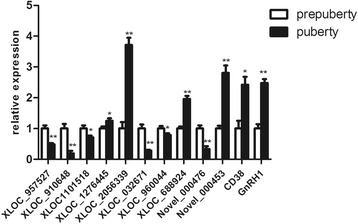
Validation of RNA-seq results by using quantitative qRT-PCR. Some lncRNAs and target genes were examined using quantitative qRT-PCR. The data are expressed as the mean ± 1 SD (*n* = 3). *p < 0.05, **p < 0.01

Further analysis identified 1165 significant differential expression transcripts (including lncRNAs, mRNAs, and novel transcripts) (*P* < 0.05), 770 up-regulated and 395 down-regulated transcripts (Fig. 3), including 57 novel transcripts. Furthermore, we detected 59 lncRNAs transcripts from 58 lncRNAs gene loci significant differentially expressed lncRNAs (*P* < 0.05), including 29 up-regulated and 30 down-regulated lncRNAs transcripts in pubertal samples compared with prepubertal samples (Additional file 2). We validated sequencing results using qRT-PCR analysis (Fig. 4).

### Prediction of target genes of lncRNAs in *cis* and *trans*

LncRNAs can act on target genes, either in *cis* (neighbor the site of lncRNA production) or in trans to coexpression whith target genes [37]. To explore whether differences in lncRNAs affects functional regulation of goat puberty, we predicted the target genes of lncRNA using the *cis* and *trans* model. To analyze the *cis* role of lncRNA, we screened protein-coding genes as potential targets 10 K/100 K upstream and downstream of the lncRNAs. The results indicated that there were 2012 lncRNAs that corresponded to 5412 target genes (Additional file 3). Interestingly, we observed several genes related puberty such as *PRLHR*, *EMC3*, *IGF2BP1*, *ZNF17*5, and *ZNF444* [38–41], which were respectively a target of *XLOC_1486935*, *XLOC_1284300*, *XLOC_957527*, *XLOC_080674*, and *XLOC_910648*, indicating that the onset of puberty is probably regulated by the lncRNA-tatget genes. Regarding the *trans* role of lncRNA, our results showed that the lncRNA, *XLOC_957527*, acted on *GnRH1* (Table 2; Additional file 4).

**Table 2:** LncRNAs and its potential target genes associated with puberty

Target genes	Cis-lncRNA	Trans-lncRNA
ZNF175	XLOC_080674	XLOC_1594657, XLOC_720006, XLOC_1831092,
DNAJB2	XLOC_1101518	XLOC_1891931, XLOC_1516990, XLOC_1554449, XLOC_1021724
EMC3	XLOC_1284300	XLOC_1429620, XLOC_1269384, XLOC_047864, XLOC_1409381, XLOC_1988116, XLOC_1430952
PRLHR	XLOC_1486935	XLOC_559241
MEF2C	XLOC_2123676	XLOC_674601, XLOC_2253794, XLOC_2334337, XLOC_1598694, XLOC_1334265, XLOC_2423839 XLOC_1178955, XLOC_1959074, XLOC_263620, XLOC_1375793
ZNF444	XLOC_910648	XLOC_1529384, XLOC_083680, XLOC_1561175, XLOC_1876674, XLOC_2092234, XLOC_1371300, XLOC_1341798, XLOC_471078, XLOC_904488, XLOC_1248122, XLOC_070767, XLOC_1753539 XLOC_1985452, XLOC_1680696, XLOC_912734, XLOC_2285546, XLOC_1011371
IGF2BP1	XLOC_957527	XLOC_1787362, XLOC_1068007, XLOC_428323, XLOC_1405012, XLOC_395262, XLOC_1970958, XLOC_228837
CD38		XLOC_523219
GnRH1		XLOC_957527

### GO and KEGG analysis

Our GO analysis of predicted targets demonstrated 73 significantly enriched terms (*P* < 0.05). The top eight terms were as follows: pheromone receptor activity, hyalurononglucosaminidase activity, hexosaminidase activity, sensory perception of taste, viral genome packaging, helicase activity, sensory perception of chemical stimulus, and sensory perception (Additional file 5). Interestingly, the signaling pathway of the pheromone receptor activity was significantly enriched, which relates to goat estrus. In addition, the sterol biosynthetic process signaling pathway was significantly enriched. *DNAJB2* was the differentially expressed target gene on the pathway, which suggests that it may be a new gene involved in the regulation of puberty onset via the sterol biosynthetic process signaling pathway.

**Fig. 5:**
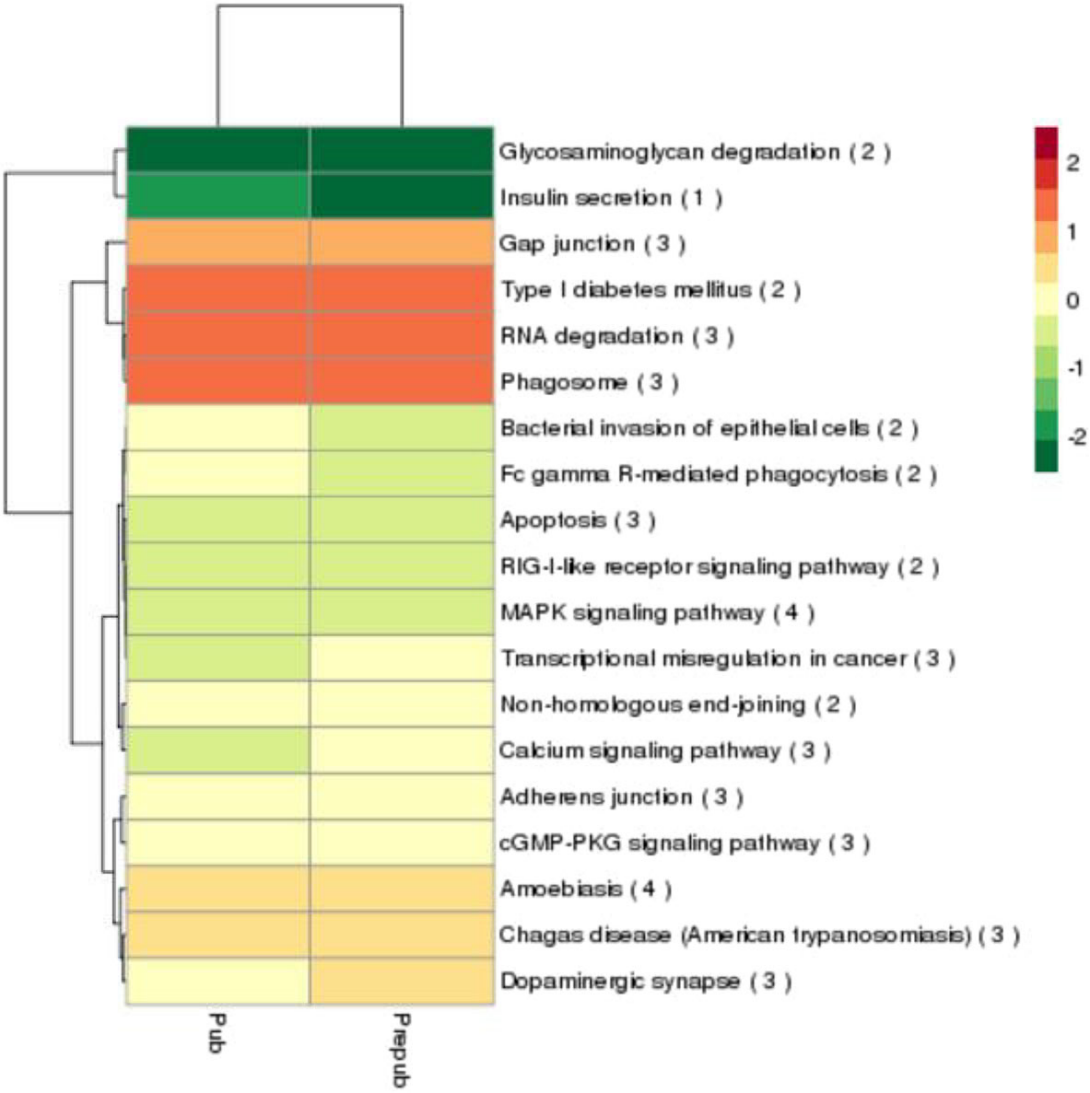
KEGG annotation for target gene functions of predicated lncRNAs. Red indicates higher expression and green indicates lower expression. The number of differentially expressed genes is shown in parentheses

KEGG pathway analysis of lncRNAs targets showed 90 terms were enriched (Additional file 6), in which oxytocin signaling pathway was related to puberty [42]. These results suggested that lncRNAs may be *cis*-acting on its target genes to regulate onset of puberty (Fig. 5).

We also evaluated the *trans* role of 2943 lncRNAs in protein-coding genes by its correlation coefficient of gene expression (Pearson correlation ≥ 0.95 or ≤ −0.95). The results showed that 2551 lncRNAs had interactions with target genes in *trans* of the goat genome. Functional analysis illustrated that target genes in *trans* were enriched (*P* < 0.05) in 158 GO terms including a variety of processes (Additional file 7), such as G-protein coupled receptor activity, transmembrane signaling receptor activity, receptor activity, and so on.

We identified 273 KEGG pathways (Additional file 8), several of which were associated with puberty, including ovarian steroidogenesis, GnRH signaling pathway, steroid biosynthesis, steroid hormone biosynthesis, oxytocin signaling pathway, GABAergic synapse, estrogen signaling pathway, oocyte meiosis, glutamatergic synapse, and others. These findings indicate that lncRNAs may act on the target genes associated with puberty of goat in *trans*.

### Specific expression of lncRNAs

There were 187 specific expressions of lncRNAs in the pubertal samples, especially, *XLOC_2409732*, which has a lower *P* than other specific expression of lncRNAs. The targets of *XLOC_2409732* were detected as *ASB5*, *WDR17*, *SPATA4* and *SPCS3* according to RNA-seq analysis. We found 243 specific expressions of lncRNAs in prepubertal samples, particularly *XLOC_1498149*, which has a lower *P* than other specific expression of lncRNAs, and *CDR1* was targeted to *XLOC_1498149* (Additional file 9). These two specifically expressed lncRNAs may play a pivotal role in goat puberty and, with further studies, provide crucial information regarding the regulation of puberty.

## Discussion

We initially performed RNA-seq to analyze lncRNAs of hypothalamus from pubertal and prepubertal female Anhuai goats. Through sequencing, we acquired 2943 predicted lncRNAs and 30162 coding transcripts. Many studies have indicated that lncRNAs have unique features compared with mRNA; for example, lncRNAs are shorter in length than protein-coding transcripts [27]. Furthermore, we found that lncRNAs in hypothalamus were shorter than in skin (1809 bp on average); however, the number of exons is similar [20]. Interestingly, the length of lncRNAs in goat hypothalamus are longer than that in human (1 kb on average) and mouse (550 nt on average), containing fewer exons than human (2.9 exons on average) and mouse (3.7 exons on average) [43].

In this study, we screened out significant differentially expressed transcripts, including 59 lncRNA transcripts from 58 lncRNA gene loci. As previous research, the functions of lncRNAs were reflected by acting on the protein-coding genes. For example, in a recent study, a muscle-specific lncRNA, *linc-MD1*, influenced muscle development by targeting to *MAML1* [44]. Moreover, the lncRNA, *Neat1*, could make a difference in pregnancy by acting on corpus luteum formation in mice [45]. Therefore, we could predict the role of mammalian lncRNAs by the relevant protein-coding genes.

Here, we predicted the potential functions of lncRNAs through the protein-coding genes in *cis* and *trans*. Several genes have been confirmed to be associated with puberty onset, including *Kiss1/GPR54* [46–49], *IGFs* [50], *GABA* [51] and *FSHR*. We discovered several differentially expressed targets in *cis* and *trans* for lncRNAs in pubertal and prepubertal hypothalamus. *PRLHR*, *EMC3*, *IGF2BP1*, *ZNF175*, *ZNF444* have been reported involved in the regulation of puberty and reproduction of animal [40, 52]. For example, puberty of female rats is significantly advanced by GnRH release under the stimulation of IGF-1; IGF-1 can affect the puberty-related events by hypothalamic GnRH release [53].

Moreover, previous research has demonstrated that puberty is delayed after over expression of ZNFs in the arcuate nucleus (ARC) of female rats; subsequent oestrous cyclicity is also disrupted [40]. Our results also showed the lncRNA, *XLOC_957527*, acted on *GnRH1* through *trans* interactions. Consequently, we confirmed that relevant lncRNAs might play a crucial role on regulation of puberty via the above targets. However, these predicted functions of lncRNAs need further experimental verification.

In the present study, oxytocin signaling pathways were enriched in KEGG pathways. *MEF2C*, as the target gene of lncRNA *XLOC_2123676*, is an important gene in oxytocin signaling pathway associated with puberty regulation. The age that vaginal opening occurs in female rats is delayed by treatment with an oxytocin antagonist, indicating that oxytocin enhances sexual maturation [42]. We also observed that *DNAJB2*, the target of lncRNA *XLOC_1101518*, has a crucial role in sterol biosynthetic process, which is involved in regulation puberty. ESR1 is essential for multiple estrogen feedback loops and required for puberty onset in female mouse [54].

**Fig. 6:**
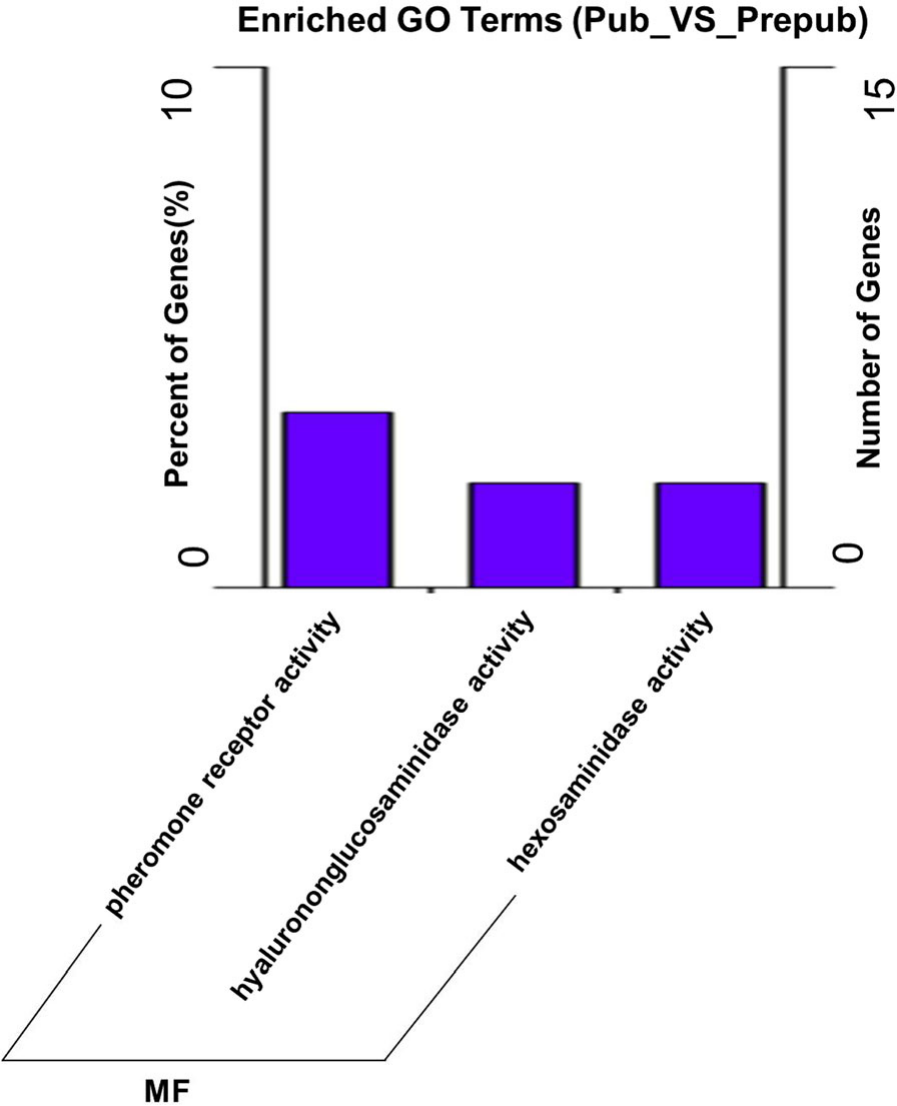
GO enrichment analysis for target gene functions of predicated lncRNAs. (MF: molecular function)

Our GO analysis of the predicted targets indicates that pheromone receptor activity signaling pathway, which relates to goat estrus, is significantly enriched (Fig. 6) [55].

In our study, the enriched KEGG pathways and GO pathways associated with reproduction and puberty clearly suggest that these lncRNAs play a vital role in regulation of puberty in goats. However, the functions of lncRNAs and their predicted targets analyses should be carefully evaluated by further experiments.

## Conclusion

We performed RNA-seq analysis, and screened out differentially expressed lncRNAs of pubertal and prepubertal goats. We elucidated genomic differences between lncRNAs compared with mRNA. Then, we observed several target genes of lncRNAs related to puberty. Our results clearly demonstrate that lncRNAs play an important role in regulating puberty in goats.

## Additional files

Additional file 1: The production of reads from the Illumina HiSeq 4000 platform. (XLSX 9 kb)
Additional file 2: The FPKM of differential expression transcripts. (XLSX 5267 kb)
Additional file 3: The protein-coding genes as potential targets 10K/100K upstream and downstream of the lncRNAs. (XLSX 372 kb)
Additional file 4: The expression of protein-coding genes related puberty. (XLSX 10 kb)
Additional file 5: GO analysis of predicted targets of lncRNAs in cis. (XLS 119 kb)
Additional file 6: KEGG pathway analysis of predicted targets of lncRNAs in cis. (XLS 21 kb)
Additional file 7: GO analysis of predicted targets of lncRNAs in trans. (XLS 1551 kb)
Additional file 8: KEGG pathway analysis of predicted targets of lncRNAs in trans. (XLS 367 kb)
Additional file 9: The specific expression of lncRNAs in pubertal/prepubertal samples. (XLSX 36 kb)

## Abbreviations

ASB5: ankyrin repeat and SOCS box containing 5
DNAJB2: DnaJ (Hsp40) homolog, subfamily B, member 2
EMC3: ER membrane protein complex subunit 3
FSHR: follicle stimulating hormone receptor
GnRH: gonadotropin-releasing hormone
IGF2BP1: insulin-like growth factor 2 mRNA binding protein 1
LncRNA: long noncoding RNA
MEF2C: myocyte enhancer factor 2C
PRLHR: prolactin releasing hormone receptor
WDR17: WD repeat domain 17
ZNF175: zinc finger protein 175
ZNF444: zinc finger protein 444
